# Restricted carbohydrate diets below 45% energy are not associated with risk of mortality in the National Health and Nutrition Examination Survey, 1999–2018

**DOI:** 10.3389/fnut.2024.1225674

**Published:** 2024-02-05

**Authors:** Austin Angelotti, Corina Kowalski, LuAnn K. Johnson, Martha A. Belury, Zach Conrad

**Affiliations:** ^1^Department of Physiology and Cell Biology, College of Medicine, The Ohio State University, Columbus, OH, United States; ^2^College of Arts and Sciences, Williamsburg, VA, United States; ^3^Independent Contractor, Warren, MN, United States; ^4^Department of Food Science and Technology, The Ohio State University, Columbus, OH, United States; ^5^Department of Kinesiology, Williamsburg, VA, United States; ^6^Global Research Institute, Williamsburg, VA, United States

**Keywords:** NHANES, restricted carbohydrate, mortality, cardiometabolic disease, cardiovascular disease, saturated fat, monounsaturated fat, polyunsaturated fat

## Abstract

**Introduction:**

Cardiometabolic diseases (CMD) are the leading causes of death for people living in the United States. Dietary strategies, such as restricting carbohydrate intake, are becoming popular strategies for improving health status. However, there is limited and often contradictory evidence on whether restricting carbohydrate intake is related to all-cause, CMD, or cardiovascular disease (CVD) mortality.

**Methods:**

The objective of the present study was to evaluate the association between restricted carbohydrate diets (<45%en) and mortality from all-causes, CMD, and CVD, stratified by fat amount and class. Data were acquired using the National Health and Nutrition Examination Survey (1999–2018) linked with mortality follow-up until December 31, 2019 from the Public-use Linked Mortality Files. Multivariable survey-weighted Cox proportional hazards models estimated hazard ratios for 7,958 adults (≥20 y) that consumed <45%en from carbohydrates and 27,930 adults that consumed 45-65%en from carbohydrates.

**Results:**

During the study period a total of 3,780 deaths occurred, including 1,048 from CMD and 1,007 from CVD, during a mean follow-up of 10.2 y. Compared to individuals that met carbohydrate recommendations (45-65%en), those that consumed carbohydrate restricted diets (<45%en) did not have significantly altered risk of mortality from all-causes (HR: 0.98; 95% CI: 0.87, 1.11), CMD (1.18; 0.95, 1.46), or CVD (1.20; 0.96, 1.49). These findings were maintained when the restricted carbohydrate diet group was stratified by intake of total fat, saturated fat (SFA), monounsaturated fat (MUFA), and polyunsaturated fat (PUFA).

**Discussion:**

Carbohydrate restriction (<45%en) was not associated with mortality from all-causes, CVD, or CMD. Greater efforts are needed to characterize the risk of mortality associated with varied degrees of carbohydrate restriction, e.g., low (<26%en) and high (>65%en) carbohydrate diets separately.

## 1 Introduction

Cardiometabolic diseases (CMD), which include heart disease, stroke, and diabetes, are the leading causes of death for men and women in the United States (1). While mortality from cardiovascular diseases (CVD) has declined over the past 20 years, mortality from endocrine and metabolic diseases has increased (2, 3). Modifiable lifestyle factors, including a healthy diet, can help mitigate the risk of mortality associated with CMD by over 60% (4).

The Acceptable Macronutrient Distribution Range (AMDR) for carbohydrates is 45–65% of total calories (5). Despite this recommendation, consumers report adopting restricted carbohydrate diets (<45%en from carbohydrates) because of their perceived health benefits (6). However, research remains inconsistent on whether restricted carbohydrate diets impact mortality risk (7–9). Some studies suggest that adopting a low carbohydrate diet elevates mortality risk due to decreased intake of fiber, fruits, and vegetables, or increased consumption of animal products and saturated fats (10, 11). Conversely, other studies suggest that a low carbohydrate diet reduces mortality risk by improving insulin sensitivity (potentially influenced by changes in hormones such as the insulin-sensitizing adipokine adiponectin) or by inhibiting the longevity-related protein, the mammalian target of rapamycin (mTOR) (12, 13).

One meta-analysis of cohort studies showed that participants that consumed lower carbohydrate diets had a 31% greater risk of all-cause mortality compared to those that consumed more carbohydrates, though no association with CVD-mortality was observed (14). A separate meta-analysis of 281 observational studies demonstrated that higher carbohydrate intake was associated with a 19% greater risk of all-cause mortality (15). Furthermore, while several nationally representative studies have observed both positive and negative associations of varied carbohydrate intake with mortality from all-causes (7, 8, 16–23) and CVD (17–21, 23, 24), none have evaluated CMD mortality. Without a clear consensus, there is a need for more high-quality studies to assess whether carbohydrate restriction is associated with reduced risk of mortality from all-causes, CMD, and CVD.

The proportion of energy derived from fat increases when carbohydrates are restricted in the diet. Dietary fat quality is known to modify the risk of all-cause and CVD mortality (25–27). Saturated fat (SFA) intake is often associated with an increased risk of all-cause and CVD mortality, while polyunsaturated fat (PUFA) is associated with a decreased risk of both (25, 27). Yet, few studies have considered dietary fat quality when studying associations between restricted carbohydrate diets and mortality.

To address these research gaps and to further inform dietary recommendations, the present study used 20 y of dietary data from the National Health and Nutrition Examination Survey (NHANES) to examine associations between restricted carbohydrate diets and risk of mortality from all-causes, CMD, and CVD. We further stratified participants consuming restricted carbohydrate diets by intake of total fat, SFA, monounsaturated fat (MUFA), and PUFA to explore whether fat quality alters the associations between carbohydrate restriction and mortality.

## 2 Methods

### 2.1 Data acquisition

Data on individual-level nutrient intake from foods and supplements, food intake, medication use, health behaviors, prevalent health conditions, family history of health conditions, and sociodemographics were acquired from NHANES, 1999–2018. NHANES collects data from approximately 5,000 non-institutionalized participants per year using a multi-stage sampling design (28). Data are collected by trained staff using in-person surveys, physical examinations, and laboratory tests. Some demographic groups are oversampled to increase reliability and precision (28). Data are collected continuously but released in two-year cycles. Dietary data are collected from participants by trained staff using an in-person 24-h dietary recall, and approximately 80% of the sample completes a second recall 3–10 days later by telephone. The computer-assisted Automated Multiple Pass Method (AMPM) is used to minimize respondent burden and increase reliability and validity (29, 30). The present study is a secondary analysis of publicly available and de-identified data and was deemed exempt from human studies ethical review by the Institutional Review Board at William & Mary. Pre-registration for this study can be found elsewhere (31).

### 2.2 Diet categorization

Usual food and nutrient intake was estimated using the National Cancer Institute’s (NCI) usual intake methodology (32). This method estimates within-person variation of the entire sample using data from two 24-h recalls collected from most participants using the SAS macros MIXTRAN, DISTRIB, and INDIVINT provided by NCI (33–35). A nonlinear mixed effects model is fit to repeated 24-h recalls using MIXTRAN. These parameter estimates are passed to DISTRIB to estimate the usual intake distribution in the population, and to INDIVINT to estimate predicted intakes at the individual level. Participants were primarily categorized into one of two groups, restricted carbohydrate (<45%en) (36) and those that met the AMDR for carbohydrate intake (45–65%en) (37), which are consistent with the categorizations used by the Nutrition and Lifestyle Task Force of the National Lipid Association (36) and the Food and Nutrient Board of the Institute of Medicine, National Academy of Sciences (Dietary Reference Intakes) (37). To investigate the effects of fat amount and class, restricted carbohydrate intakes were further stratified by intake tertiles of total fat (<35.5%en, 35.5–38.3%en, >38.3%en), SFA (<11.3%en, 11.3–12.4%en, >12.4%en), MUFA (<12.7%en, 12.7–13.8%en, >13.8%en), and PUFA (<7.6%en, 7.6–8.7%en, >8.7%en).

### 2.3 Outcome ascertainment

Participants from NHANES were linked to the Public-use Linked Mortality Files (1999–2019) (38), which provide mortality follow-up through December 31, 2019, the latest date available. Follow-up was defined as the time from NHANES data collection to death or December 31, 2019, whichever came first. Standardized procedures were used by the National Center for Health Statistics (NCHS) to adjudicate deaths. Staff at NCHS used probabilistic matching to link NHANES participants to records in the National Death Index (NDI) using identifying information such as social security number, name, date of birth, and state of residence (39). The NDI records all US deaths since 1979, and includes nearly all deaths (~97%) when social security numbers are available, such is the case for all eligible NHANES participants linked to the NDI (40, 41).

Mortality follow-up data for NHANES participants were collected for all causes, coronary heart disease (International Classification of Disease 10th revision codes I00–I09, I11, I13, I20–I51), stroke (I60–I69), and diabetes (E10–E14). Deaths from coronary heart disease and stroke were summed to represent deaths from CVD, and deaths from CVD and diabetes were summed to represent deaths from CMD. Deaths from heart disease, stroke, and diabetes were evaluated as part of CVD and CMD, but were not evaluated as separate outcomes due to insufficient sample sizes (829, 178, 41 cases, respectively).

### 2.4 Covariates

Data on self-reported age, sex, education, and race/ethnicity were acquired from the demographic questionnaire. NHANES staff used self-reported information on household income and household composition (number of persons including children) to calculate income-to-poverty ratio (ratio of household income compared to the federal poverty guideline). Data on physical activity were acquired from the physical activity questionnaire, and data from 1999–2006 and 2007–2018 were harmonized using the approach by Bailey et al. (42). Data on smoking status were acquired from the questionnaire on recent tobacco use. NHANES staff collected height and weight measurements and used this information to calculate Body Mass Index (BMI; kg/m2). NHANES staff measured systolic and diastolic blood pressure at the brachial artery after participants were seated and resting, and the average of ≥2 measurements were calculated for each participant, which is consistent with clinical standards (43). Hypertension was determined by systolic blood pressure ≥ 130 mmHg or diastolic blood pressure ≥ 80 mmHg or self-report of current prescription drug treatment (43). Lipid panels were used to collect information on serum triglycerides and high-density lipoprotein cholesterol (HDL-C). Participants with triglyceride levels ≥150 mg/dL (≥1.695 mmol/L) or self-report of current prescription drug treatment were identified as having elevated triglycerides, and those with HDL-C < 50 mg/dL (<1.295 mmol/L) for women or < 40 mg/dL (<1.036 mmol/L) for men or self-report of current prescription drug treatment were identified as having low HDL-C (44). Participants with elevated triglycerides and low HDL-C were classified as having dyslipidemia (44). Baseline heart disease, stroke, and diabetes were evaluated using standard measures from the American Heart Association (45), which include self-report of physician diagnosis, prescription drug treatment, fasting plasma glucose, and the Rose questionnaire (undiagnosed angina), described elsewhere (46). Data on daily intake of energy, protein, and alcohol were acquired from the 24-h recall files. Data on daily intake of refined grains and added sugar were acquired from the Food Patterns Equivalents Database, which converts data on food intake from 24-h recalls into food group equivalents (47).

### 2.5 Statistical analyses

Multivariable Cox proportional hazards regression models assessed the association between diet patterns and mortality from all causes, CMD, and CVD. Base models were adjusted for demographic variables, health behaviors, and baseline health status including age (20–30 y, 31–50 y, 51–70 y, or ≥ 71 y), sex (male or female), education (<high school, high school or equivalent, some college, or college graduate), race-ethnicity (non-Hispanic white, non-Hispanic black, Mexican-American, or other), income-to-poverty ratio (<0.75, 0.75–1.30, 1.31–1.99, 2.00–3.99, or ≥ 4.00), physical activity (sedentary, moderate, or vigorous) (42), smoking status (<100 cigarettes in lifetime, ≥100 cigarettes in lifetime but not current smoker, ≥100 cigarettes in lifetime and currently smoke some days, or ≥ 100 cigarettes in lifetime and currently smoke everyday), BMI (<18.5, 18.5- < 25, 25- < 30, or ≥ 30), baseline hypertension (yes or no), baseline dyslipidemia (yes or no), family history of heart disease (yes or no), family history of diabetes (yes or no), and survey cycle (continuous). Missing values for each covariate were included as a dummy indicator to preserve sample size.

Fully adjusted models were adjusted for dietary factors including daily intake of energy (kcal, continuous), refined grains (oz. equivalents, continuous), added sugar (tspn. Equivalents, continuous), fiber (g, continuous), protein (%en, continuous), and alcohol (%en, continuous). Models stratified by total fat intake were also adjusted for unsaturated-to-saturated fat ratio, models stratified by SFA intake were additionally adjusted for MUFA (%en, continuous) and PUFA (%en, continuous), models stratified by MUFA intake were additionally adjusted for SFA (%en, continuous) and PUFA (%en, continuous), and models stratified by PUFA intake were additionally adjusted for SFA (%en, continuous) and MUFA (%en, continuous). Supplemental models adjusted for additional dietary factors that may have a neutral or beneficial effect on CMD (48). Models stratified by SFA intake were additionally adjusted for stearic acid, models stratified by MUFA intake were additionally adjusted for oleic acid, and models stratified by PUFA intake were additionally adjusted for linoleic acid, α-linolenic acid, eicosapentaenoic acid, and docosahexaenoic acid.

Statistical significance was set at *p* < 0.05 with Bonferroni adjustment for multiple comparisons. Standard errors were estimated with the balanced repeated replication method while accounting for the multistage probability sampling design of NHANES. Stata 16.1 (StataCorp; College Station, TX) (49) was used for data management and SAS 9.4 (SAS Institute; Cary, NC) (50) was used for all analyses.

## 3 Results

### 3.1 Participant characteristics

A total of 96,766 individuals provided dietary data from 1999–2018 (Supplementary Figure S1). Participants were excluded if they were < 20 y of age (*n* = 44,368); pregnant or breastfeeding (*n* = 1,827); had incomplete or unreliable dietary recalls as deemed by trained NHANES staff (*n* = 3,570); were unable to be linked to the National Death Index due to incomplete identifying information (*n* = 81); died during the first year of follow up (*n* = 468); had heart disease, stroke, or diabetes at baseline (*n* = 10,542); or consumed >65%en from carbohydrates (*n* = 22). A total of 35,888 US participants were included in the analytic sample. During the study period a total of 3,780 deaths occurred, including 1,048 from cardiometabolic disease (CMD), and 1,007 from cardiovascular disease (CVD).

Table 1 describes participant characteristics for those consuming 45-65%en carbohydrate (recommended carbohydrate) and those consuming <45%en carbohydrate (restricted carbohydrate). Mean carbohydrate intake was 41%en for the restricted carbohydrate group and 50.4%en for the recommended carbohydrate group (*p* < 0.001). Accordingly, the restricted carbohydrate group had higher mean fat and protein intakes (37%en fat, 16%en protein) than the recommended carbohydrate group (34%en fat, 15%en protein; *p* < 0.001 for both comparisons). Mean alcohol intake was also higher among the restricted carbohydrate group (7%en) compared to the recommended carbohydrate group (4%en; *p* < 0.001).

**Table 1:** Participant characteristics, National Health and Nutrition Examination Survey, 1999–2018 (*n* = 35,888).

	Recommended carbohydrate^a^	Restricted carbohydrate^a^	*p*-value^b^
	(*n* = 27,930)	(*n* = 7,958)	
Energy (kcal)	2,211 (2,203 to 2,221)	2,217 (2,200 to 2,234)	0.574
Macronutrient intake			
Carbohydrate intake (%en)	50.4 (50.4 to 50.5)	41.3 (41.2 to 41.4)	<0.001
Fat intake (%en)	33.5 (33.4 to 33.5)	36.6 (36.5 to 36.8)	<0.001
Protein intake (%en)	15.1 (15.1 to 15.1)	16.3 (16.2 to 16.3)	<0.001
Alcohol intake (%en)	3.5 (3.4 to 3.6)	7.1 (6.9 to 7.3)	<0.001
Age (mean years)	44.4 (44.0 to 44.7)	44.9 (44.4 to 45.5)	0.036
Income-to-poverty ratio (mean)	3.0 (2.9 to 3.0)	3.4 (3.3 to 3.5)	<0.001
BMI (mean kg/m2)	28.1 (27.9 to 28.2)	28.7 (28.5 to 29.0)	<0.001
Female (%)	53.6 (52.8 to 54.4)	44.5 (43.0 to 46.0)	<0.001
Race/ethnicity (%)			<0.001
Non-Hispanic white	66.9 (64.7 to 69.0)	74.7 (72.7 to 76.6)	
Non-Hispanic black	11.0 (9.9 to 12.1)	10.2 (9.1 to 11.3)	
Mexican American	9.0 (7.9 to 10.2)	6.2 (5.3 to 7.4)	
Other	13.2 (12.1 to 14.4)	8.9 (8.0 to 9.9)	
Education (%)			<0.001
Less than high school	16.4 (15.6 to 17.3)	11.8 (10.8 to 12.9)	
High school or equivalent	24.5 (23.5 to 25.5)	21.4 (20.2 to 22.7)	
Some college	31.5 (30.6 to 32.4)	32.5 (30.9 to 34.0)	
College graduate	27.5 (26.1 to 29.0)	34.2 (31.9 to 36.6)	
Physical activity (%)			<0.001
Sedentary	25.0 (24.1 to 26.0)	20.9 (19.6 to 22.2)	
Moderate	33.5 (32.5 to 34.4)	32.4 (30.9 to 33.9)	
Vigorous	41.5 (40.2 to 42.8)	46.7 (45.1 to 48.4)	
Smoking status (%)			<0.001
<100 lifetime cigarettes	57.5 (56.4 to 58.6)	47.8 (46.0 to 49.6)	
>100 lifetime cigarettes, current non-smoker	21.4 (20.6 to 22.2)	26.6 (25.2 to 28.0)	
>100 lifetime cigarettes, smokes some days	3.7 (3.4 to 4.1)	5.4 (4.7 to 4.1)	
>100 lifetime cigarettes, every day smoker	17.3 (19.0 to 21.6)	20.2 (16.4 to 18.2)	
Hypertension^a^			0.007
Yes	43.3 (42.4–44.2)	46.2 (44.7–47.7)	
No	56.7 (55.8–57.6)	53.8 (52.3–55.3)	
Dyslipidemia^b^			0.323
Yes	13.7 (13.0–14.4)	13.5 (12.4–14.6)	
No	35.5 (34.5–36.5)	36.8 (35.2–38.4)	
Missing	50.8 (50.0–51.9)	49.7 (48.0–51.6)	
Mortality cases			
All causes	3,060	720	
Cardiometabolic disease^c^	846	202	
Cardiovascular disease^d^	810	197	

Values in parentheses are 95% CI.

a Systolic blood pressure ≥ 130 mmHg or diastolic blood pressure ≥ 80 mmHg or self-report of current prescription drug treatment (43).

b Elevated triglycerides (≥150 mg/dL or self-report of current prescription drug treatment) and low HDL-C (<50 mg/dL for women or < 40 mg/dL for men or self-report of current prescription drug treatment) (44).

c Heart disease, stroke, and diabetes.

d Heart disease and stroke.

The restricted carbohydrate group had a higher mean age (+0.6 years; *p* = 0.036), BMI (+0.7 kg/m2; *p* < 0.001), and income-to-poverty ratio (+0.4; *p* < 0.001) when compared to the recommended carbohydrate group. A greater proportion of participants in the restricted carbohydrate group were male (+9.1 percentage points; *p* < 0.001), non-Hispanic white (+7.8 percentage points; *p* < 0.001), college graduates (+6.7 percentage points; *p* < 0.001), participated in vigorous physical activity (+5.2 percentage points; *p* < 0.001), and smoked cigarettes every day (+2.9 percentage points; *p* < 0.001).

### 3.2 Restricted carbohydrate diet and mortality

**Figure 1:**
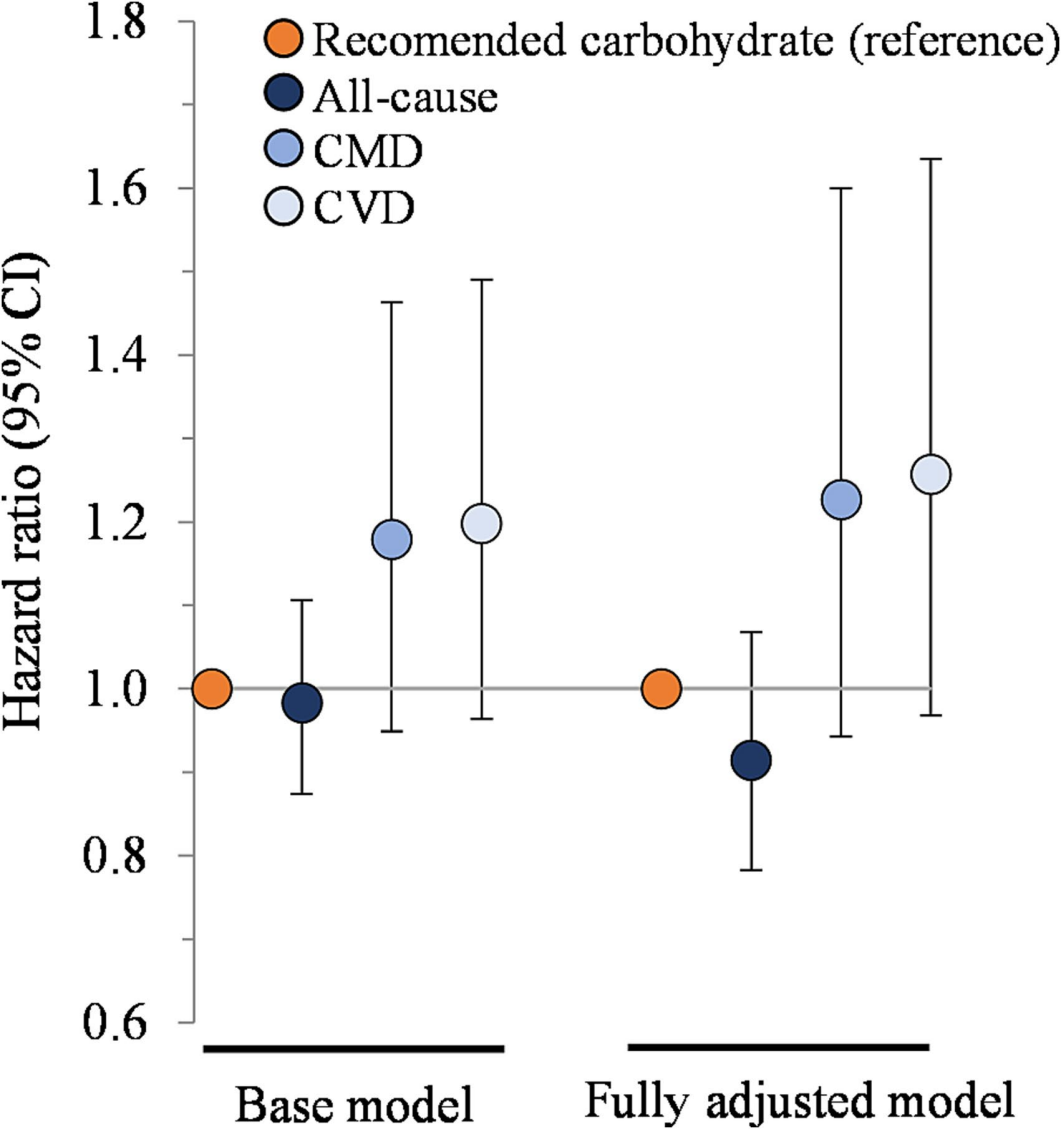
Association between carbohydrate restriction and risk of mortality from all causes, CMD, and CVD, 1999–2018 (*n* = 35,888). Hazard ratios (with 95% confidence intervals) comparing risk of mortality between carbohydrate restricted diet patterns (<45%en) to recommended carbohydrate diet patterns (45–65%en), calculated using Cox proportional hazards models. Base model: adjusted for age (y), sex, race/ethnicity, education, smoking status, income-to-poverty ratio, physical activity (level), baseline hypertension, baseline dyslipidemia, family history of heart disease, family history of diabetes, body mass index (kg/m2), and NHANES survey wave. Fully adjusted model: base model + energy (kcal), refined grains (ounce-equivalents), added sugars (tsp equivalent), fiber (grams), protein (%en), and alcohol (%en). CMD, cardiometabolic disease; CVD, cardiovascular disease; NHANES, National Health and Nutrition Examination Survey.

In the multivariable base model, hazard ratios for participants that consumed restricted carbohydrate diets were 0.98 (95% CI: 0.87, 1.11) for all-cause mortality, 1.18 (0.95, 1.46) for CMD mortality, and 1.20 (0.96, 1.49) for CVD mortality, compared to participants that met carbohydrate recommendations (Figure 1). These findings did not change upon further adjustment for dietary factors (energy, refined grains, added sugars, fiber, protein, and alcohol) in the fully adjusted model (*p* > 0.05 for all comparisons).

**Figure 2:**
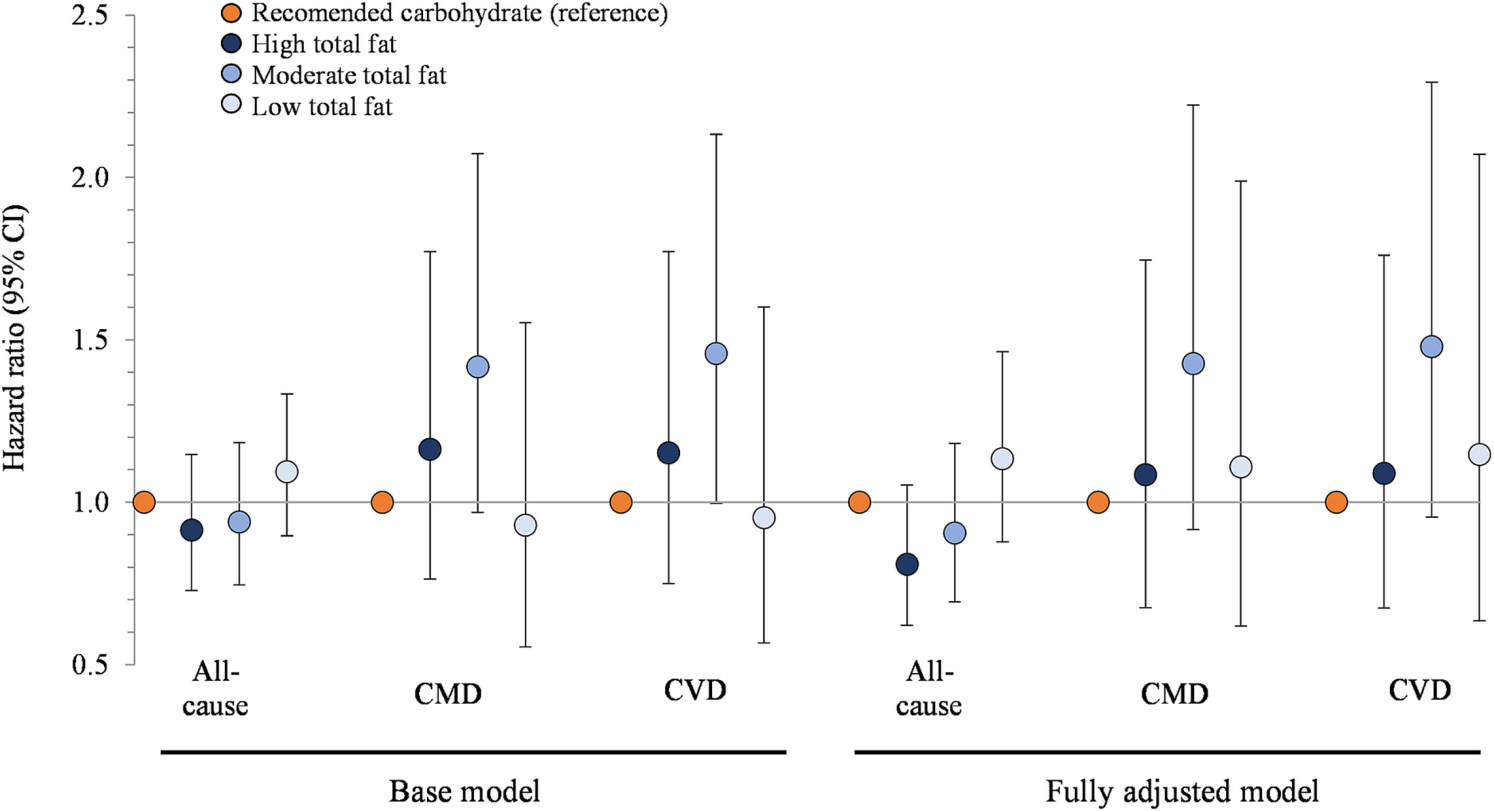
Associations between carbohydrate restriction, stratified by total fat intake, and risk of mortality from all causes, CMD, and CVD, 1999–2018 (*n* = 35,888). Hazard ratios (with 95% confidence intervals) comparing risk of mortality between carbohydrate restricted diet patterns (<45%en) to recommended carbohydrate diet patterns (45–65%en), calculated using Cox proportional hazards models. Base model: adjusted for age (y), sex, race/ethnicity, education, smoking status, income-to-poverty ratio, physical activity (level), baseline hypertension, baseline dyslipidemia, family history of heart disease, family history of diabetes, body mass index (kg/m2), and NHANES survey wave. Fully adjusted model: base model + energy (kcal), refined grains (ounce-equivalents), added sugars (tsp equivalent), fiber (grams), protein (%en), alcohol (%en), unsaturated to saturated fat ratio. CMD, cardiometabolic disease; CVD, cardiovascular disease; NHANES, National Health and Nutrition Examination Survey.

Figure 2 presents the association between carbohydrate restriction and mortality stratified by total fat intake. The hazard ratios for all-cause mortality, from the highest to the lowest tertile of fat intake, were 0.91 (95% CI: 0.73, 1.15), 0.94 (0.75, 1.18), and 1.09 (0.90, 1.33; *p* > 0.05 for all comparisons). The hazard ratios for CMD mortality, from the highest to lowest tertile of fat intake, were 1.16 (0.76, 1.77), 1.42 (0.97, 2.07), and 0.93 (0.56, 1.55; *p* > 0.05 for all comparisons), which were similar to the hazard ratios for CVD mortality. Additional adjustments for energy intake, refined grains, added sugars, fiber, protein, alcohol, and unsaturated to saturated fat ratio in the fully adjusted models did not modify these findings.

### 3.3 Restricted carbohydrate diet and mortality, stratified by fat class

**Figure 3:**
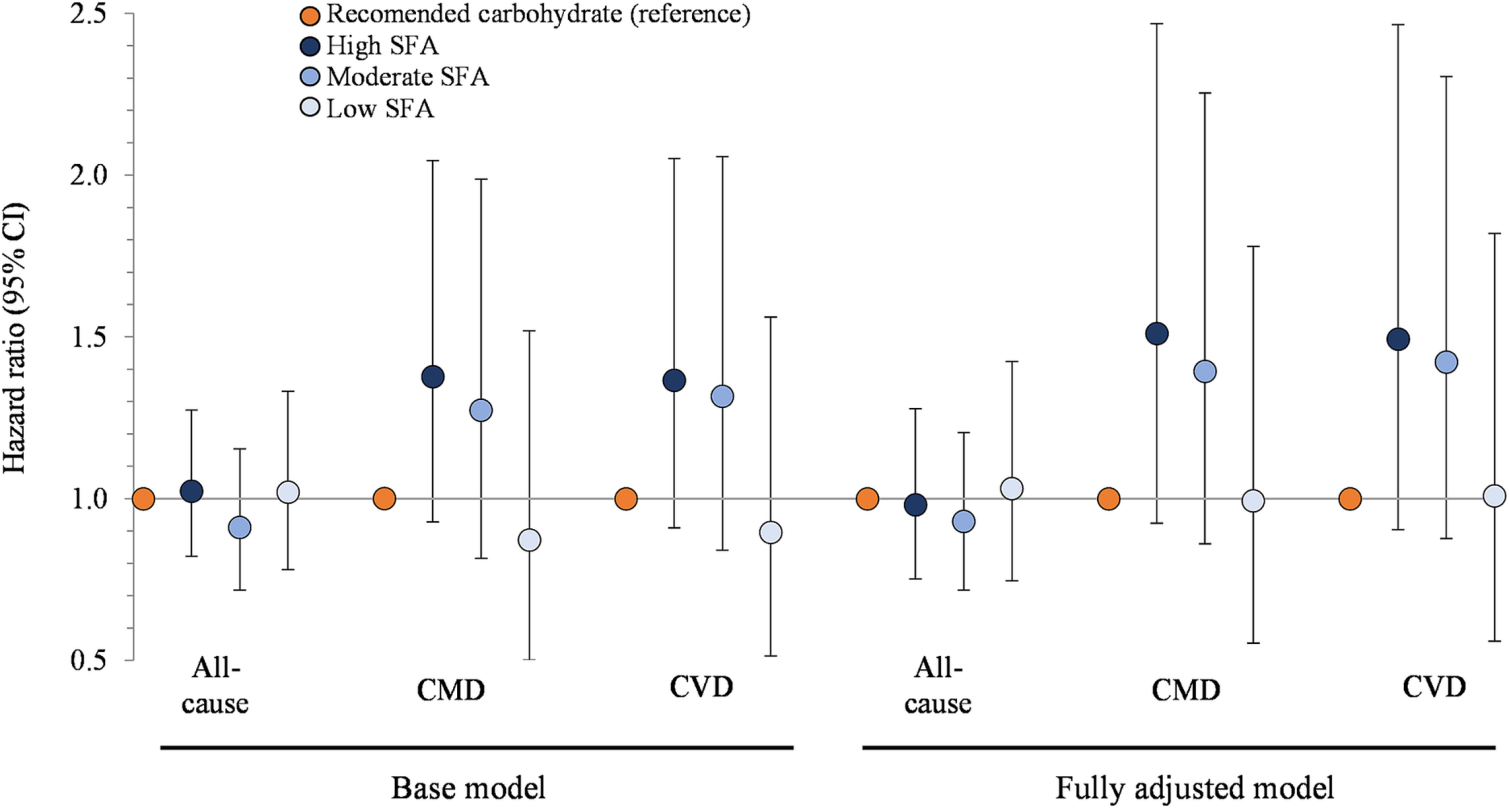
Associations between carbohydrate restriction, stratified by SFA intake, and risk of mortality from all causes, CMD, and CVD, 1999–2018 (*n* = 35,888). Hazard ratios (with 95% confidence intervals) comparing risk of mortality between carbohydrate restricted diet patterns (<45%en) to recommended carbohydrate diet patterns (45–65%en), calculated using Cox proportional hazards models. Base model: adjusted for age (y), sex, race/ethnicity, education, smoking status, income-to-poverty ratio, physical activity (level), baseline hypertension, baseline dyslipidemia, family history of heart disease, family history of diabetes, body mass index (kg/m2), and NHANES survey wave. Fully adjusted model: base model + energy (kcal), refined grains (ounce-equivalents), added sugars (tsp equivalent), fiber (grams), protein (%en), alcohol (%en), MUFA (%en), and PUFA (%en). CMD, cardiometabolic disease; CVD, cardiovascular disease; NHANES, National Health and Nutrition Examination Survey; SFA, saturated fat; MUFA, monounsaturated fat; PUFA, polyunsaturated fat.

Figure 3 presents the association between carbohydrate restriction and mortality stratified by SFA intake. Hazard ratios for all-cause mortality, from highest to lowest tertile of SFA intake, were 1.02 (95% CI: 0.82, 1.27), 0.91 (0.71, 1.15), and 1.02 (0.78, 1.33; *p* > 0.05 for all comparisons). The hazard ratios for CMD mortality, from the highest to lowest tertile of SFA intake, were 1.38 (0.93, 2.05), 1.27 (0.82, 1.99), and 0.87 (0.50, 1.52; *p* > 0.05 for all comparisons), which mirrored the hazard ratios for CVD mortality. The additional adjustments in the fully adjusted models (energy, refined grains, added sugars, fiber, protein, and alcohol), along with the adjustment for steric acid intake (Supplementary Figure S2), did not meaningfully alter the results.

**Figure 4:**
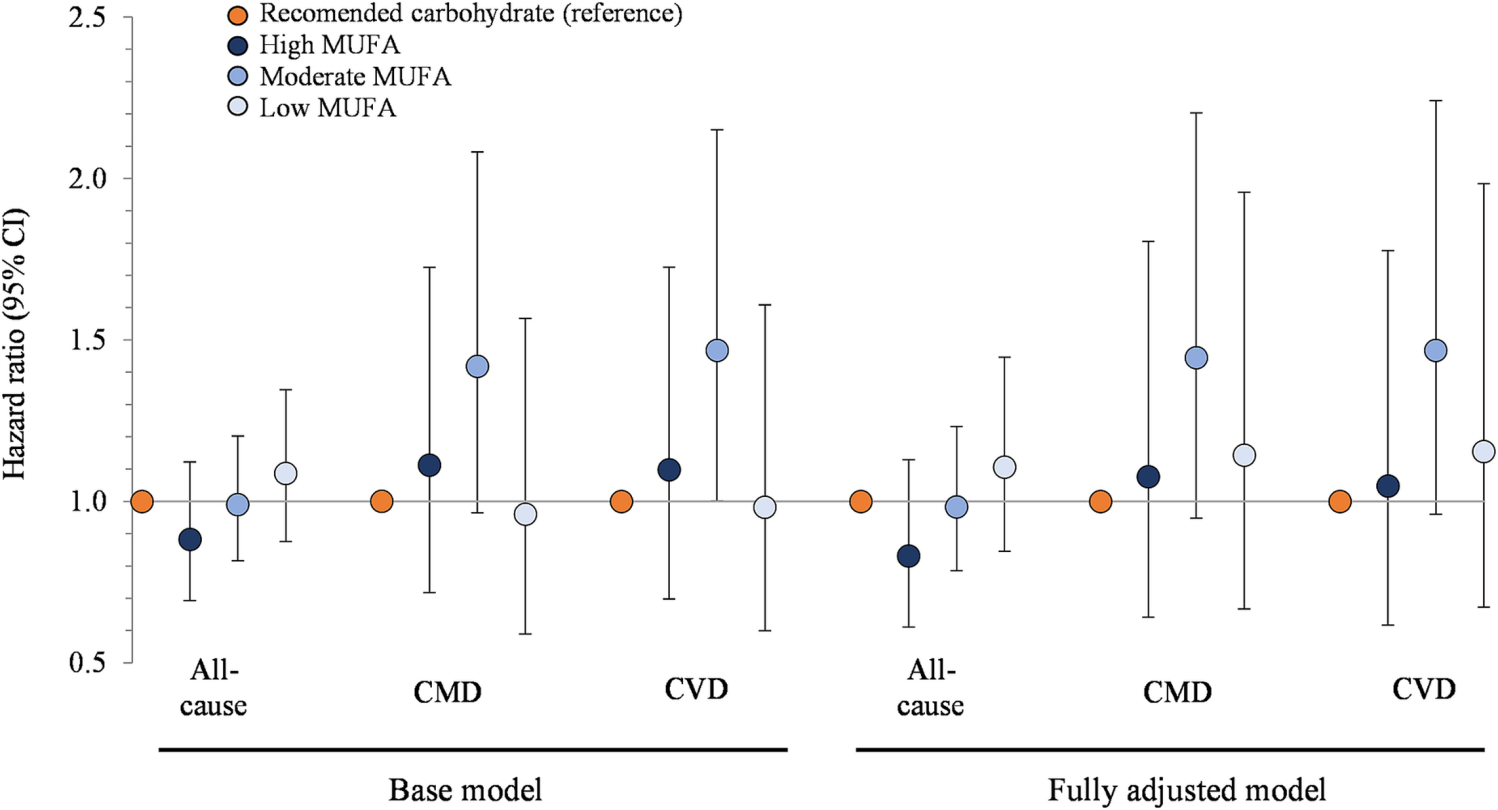
Associations between carbohydrate restriction, stratified by MUFA intake, and risk of mortality from all causes, CMD, and CVD, 1999–2018 (*n* = 35,888). Hazard ratios (with 95% confidence intervals) comparing risk of mortality between carbohydrate restricted diet patterns (<45%en) to recommended carbohydrate diet patterns (45–65%en), calculated using Cox proportional hazards models. Base model: adjusted for age (y), sex, race/ethnicity, education, smoking status, income-to-poverty ratio, physical activity (level), baseline hypertension, baseline dyslipidemia, family history of heart disease, family history of diabetes, body mass index (kg/m2), and NHANES survey wave. Fully adjusted model: base model + energy (kcal), refined grains (ounce-equivalents), added sugars (tsp equivalent), fiber (grams), protein (%en), alcohol (%en), SFA (%en), and PUFA (%en). CMD, cardiometabolic disease; CVD, cardiovascular disease; NHANES, National Health and Nutrition Examination Survey; MUFA, monounsaturated fat; SFA, saturated fat; PUFA, polyunsaturated fat.

Figure 4 shows the associations between carbohydrate restricted diets and mortality, stratified by MUFA intake. Hazard ratios for all-cause mortality, from highest to lowest tertile of MUFA intake, were 0.88 (95% CI: 0.69, 1.12), 0.99 (0.82, 1.20), and 1.09 (0.88, 1.35; *p* > 0.05 for all comparisons). The hazard ratios for CMD mortality, from the highest to lowest tertile of MUFA intake, were 1.11 (0.72, 1.73), 1.42 (0.97, 2.08), and 0.96 (0.59, 1.57; *p* > 0.05 for all comparisons). For CVD mortality, participants with moderate intakes had elevated mortality risk (1.47, 1.00–2.15; *p* = 0.049) but this became non-statistically significant in the fully adjusted model (energy, refined grains, added sugars, fiber, protein, and alcohol; *p* > 0.05) and the supplemental model additionally adjusted for oleic acid intake (*p* > 0.05; Supplementary Figure S3). For participants in the highest and lowest tertiles, hazard ratios for CVD mortality were similar to mortality from CMD and all causes, and neither the adjustments made in the fully adjusted models (energy, refined grains, added sugars, fiber, protein, and alcohol) nor the additional adjustment for oleic acid intake (Supplementary Figure S3) changed the results.

**Figure 5:**
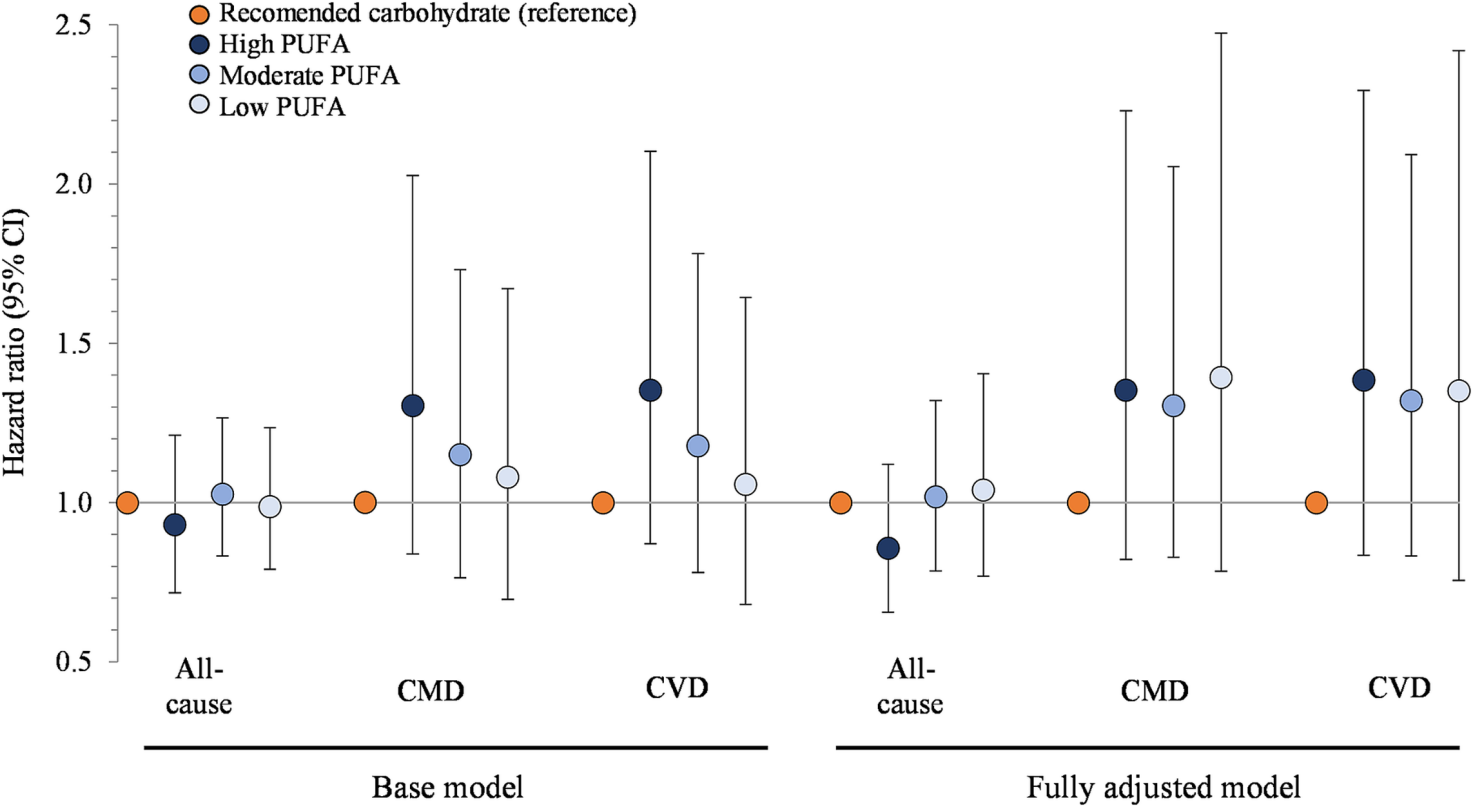
Associations between carbohydrate restriction, stratified by PUFA intake, and risk of mortality from all causes, CMD, and CVD, 1999–2018 (*n* = 35,888). Hazard ratios (with 95% confidence intervals) comparing risk of mortality between carbohydrate restricted diet patterns (<45%en) to recommended carbohydrate diet patterns (45–65%en), calculated using Cox proportional hazards models. Base model: adjusted for age (y), sex, race/ethnicity, education, smoking status, income-to-poverty ratio, physical activity (level), baseline hypertension, baseline dyslipidemia, family history of heart disease, family history of diabetes, body mass index (kg/m2), and NHANES survey wave. Fully adjusted model: base model + energy (kcal), refined grains (ounce-equivalents), added sugars (tsp equivalent), fiber (grams), protein (%en), alcohol (%en), SFA (%en), and MUFA (%en). CMD, cardiometabolic disease; CVD, cardiovascular disease; NHANES, National Health and Nutrition Examination Survey; PUFA, polyunsaturated fat; SFA, saturated fat; MUFA, monounsaturated fat.

Figure 5 presents the association between carbohydrate restriction and mortality stratified by PUFA intake. The multivariate base model hazard ratios, from the highest to lowest tertile of PUFA intake, were 0.93 (95% CI: 0.72, 1.21), 1.03 (0.83, 1.27), and 0.99 (0.79, 1.24; *p* > 0.05 for all comparisons). The hazard ratios for CMD mortality, from the highest to lowest tertile of PUFA intake, were 1.30 (0.84, 2.03), 1.15 (0.76, 1.73), and 1.08 (0.70, 1.67; *p* > 0.05 for all comparisons), which were similar to that hazard ratios for CVD mortality. Further adjustment for energy, refined grains, added sugars, fiber, protein, and alcohol in the fully adjusted model, as well as additional adjustment for intake of eicosapentaenoic acid and docosahexaenoic acid (Supplementary Figure S4), did not modify the main findings.

## 4 Discussion

In this nationally representative study of over 35,000 participants with a mean follow-up of 10.2 y, a restricted carbohydrate diet (<45%en carbohydrate) was not associated with risk of mortality from all-causes, CMD, or CVD in fully adjusted models compared to a diet that met carbohydrate recommendations (45–65%en). Stratification by fat amount and class did not alter these main findings.

To our knowledge, only two other nationally representative studies in the US have reported associations between carbohydrate intake and mortality risk, of which have conflicting results (10, 16). Mazidi et al. (10) reported that lower carbohydrate diets were associated with an elevated risk of mortality from all-causes, coronary heart disease, and stroke in a 1999–2010 sample of 24,825 NHANES participants with follow-up until 2011. A later study by Shan et al. (16) found no association between carbohydrate restriction and mortality from all causes in a 1999–2014 sample of 37,233 NHANES participants with follow-up until 2015, which is consistent with the present study. This discrepancy between prior studies is likely due to differences in assessment of dietary intake and exclusion criteria. Mazidi et al. (10) used a single day of dietary recall to evaluate acute intake rather than usual intake, and was thus subject to within-person random error and overestimates the range of dietary intakes in the population (51) (mean carbohydrate intake of extreme quartiles was 39%en and 64%en). Rather, it is recommended that longitudinal studies employ multiple dietary recalls collected from most participants to reduce measurement bias, and analyze these data using usual intake methodologies such as the NCI method (51), as did Shan et al. (16) (mean carbohydrate intake of extreme quintiles was 46 and 58%). To further control for bias, Shan et al. excluded participants with a history of heart disease or cancer, as well as those that died during the first year of follow-up (16), whereas Mazidi et al. did not (10). The methodology and results of the present study (mean carbohydrate intake of 41% in the restricted carbohydrate group and 50% in the recommended carbohydrate group) are consistent with Shan et al. (16).

Several other studies have compared carbohydrate intake and the risk of all-cause mortality in prospective cohorts, with inconsistent findings (7, 11, 18–21, 23, 52–54). Of these studies, six reported either no association between carbohydrate intake and all-cause mortality (18, 23) or a “U”-shaped association with the lowest risk of all-cause mortality within the 45-65%en range for carbohydrate intake (7, 21, 52, 53). Two studies reported a modest increase in all-cause mortality risk (HR: 1.12; 95% CI: 1.01, 1.24 and 1.06; 1.00, 1.12 respectively) with lower carbohydrate diet score (11, 20), and two studies reported a decreased risk of all-cause mortality (HR: 0.74, 0.57–0.95; 1.28, 1.12–1.46 respectively) with lower carbohydrate intake (19, 54). Interestingly, the two studies that reported lower all-cause mortality risk included participants with a large range of carbohydrate intake from 45%en to above 70%en (19, 54). This could explain the discrepancies between their results and the results reported in this study (which excluded participants that consumed above the recommended carbohydrate intake of 65%en). If individuals that consume >65%en from carbohydrates have a higher risk of all-cause mortality (as some studies suggest), study populations with higher carbohydrate intake would likely show a decreased risk of all-cause mortality with lower carbohydrate intake, while studies such as ours would not be able to detect.

Prior studies that evaluated the association between carbohydrate intake and CVD mortality have also shown mixed results (11, 19–21, 23, 24, 52, 54, 55). Several reported no association between low carbohydrate intake and CVD mortality (20, 23, 24, 54, 55). Alternatively, two prior studies reported a non-linear “U”-shaped association with CVD mortality with the lowest risk associated with 50-55%en from carbohydrates (21, 52), two studies reported a lower carbohydrate diet score resulted in an increased risk of CVD mortality in men (11, 52), and one study reported that a lower carbohydrate diet score resulted in a decreased risk of CVD mortality in women (19).

To our knowledge, no other study has evaluated the association between carbohydrate intake and CMD mortality, despite the rise of mortality from metabolic diseases over the last decade (2). In this study, the hazard ratios for CMD mortality mirrored the hazard ratios for CVD, likely because 96% of deaths from CMD were also categorized as CVD deaths. More research is needed to confirm the CMD mortality findings of the present study.

Additionally, no prior studies have evaluated the association between restricted carbohydrate diets and mortality when stratified by intake of dietary fat classes. The lack of association between fat-stratified diets in this study is surprising, given that prior evidence generally demonstrates a reduced risk of mortality (all-cause and CVD) associated with higher intake of unsaturated fats (21, 27, 56–59) and an elevated risk of mortality associated with higher intake of SFA (21, 27, 57). However, these previous studies focused on total intake of SFA, MUFA, and PUFA, while in this study only those consuming restricted carbohydrate diets (<45%en carbohydrates) were stratified by dietary fat class. It is possible that changes in dietary fat class have a greater impact on mortality at lower ranges of intake. For example, a difference in PUFA intake from 2%en to 4%en may impact mortality more than a difference from 10%en to 12%en. Because those consuming restricted carbohydrate diets had higher fat intake, changes in dietary fat class may be less impactful (or even non-existent) at these higher intakes.

Other studies have reported associations between carbohydrate intake and mortality after categorizing carbohydrate intake into “healthy” or “unhealthy” (8, 16), animal or plant-based (9), and carbohydrate type, such as starch or fiber (17, 21, 24, 55). Generally, these studies reported that consuming diets lower in “healthy” carbohydrates (8, 16), lower in vegetable-based carbohydrates (52), and lower in fiber (17, 55) increased mortality risk, while consuming diets lower in “unhealthy” carbohydrates (8, 16), lower in animal-based carbohydrates (9), and lower in starch and sugar (17, 55) decreased mortality risk. These findings suggest that carbohydrate quality impacts mortality risk more than carbohydrate quantity.

This study has several strengths. The large sample size, multistage sampling design, and survey weighting make these findings generalizable to the US population. Usual dietary intake was assessed from multiple 24-h recalls using the NCI method which reduces bias by accounting for within-person variation, measuring habitual and episodic intake of foods and nutrients, and correlating the amount consumed to the probability of intake. Modification by fat intake was assessed by evaluating fat classes independently (SFA, MUFA, and PUFA) as well as cumulatively (total fat). To further minimize bias, participants were excluded if they had died during the first year of follow up or if they had outcome measures at baseline (prevalent heart disease, stroke, or diabetes). Further, iterative models adjusted for sociodemographic factors and health behaviors (base); energy, food components, and fat classes (fully adjusted); and individual fatty acids (supplemental). This study also has limitations. NHANES samples new participants during each survey cycle (2-year period) so long-term changes in individual-level dietary intake cannot be measured. The sample sizes for participants that consumed low (<26%en) and high (>65%en) carbohydrate diets were too small to produce reliable nationally representative estimates, so future studies are needed to evaluate diet-disease relationships for these groups. Mortality from coronary heart disease, stroke, and diabetes could not be evaluated separately due to small sample sizes for these outcomes (829, 178, and 41 cases, respectively) so these were included in aggregate analyses (all-cause, CMD, and CVD). Finally, the observational design of this study precludes determination of causality.

## 5 Conclusion

In this nationally representative sample of over 35,000 individuals followed for a mean of over 10 years, those that consumed restricted carbohydrate diets (<45%en) did not have significantly different risk of mortality (from all-causes, cardiometabolic disease, or cardiovascular disease) compared to those that met carbohydrate intake recommendations (45–65%en) These findings remained consistent after stratifying by fat amount and class (saturated fat, monounsaturated fat, and polyunsaturated fat). Due to the limited number of participants that consumed <26%en and > 65%en from carbohydrates, it was not feasible to measure the mortality risk associated with these extreme intakes, emphasizing the need for future research in this area.

## Data availability statement

Publicly available datasets were analyzed in this study. This data can be found here: https://www.cdc.gov/nchs/nhanes/index.htm; https://www.cdc.gov/nchs/data-linkage/mortality-public.htm.

## Ethics statement

The studies involving humans were approved by Institutional Review Board at William & Mary. The studies were conducted in accordance with the local legislation and institutional requirements. Written informed consent for participation was not required from the participants or the participants’ legal guardians/next of kin in accordance with the national legislation and institutional requirements.

## Author contributions

ZC and MB: conceptualization, methodology, and funding acquisition. ZC and LJ: software, validation, formal analysis, investigation, and data curation. ZC: resources, supervision, and project administration. AA: writing—original draft preparation. AA, CK, LJ, MB, and ZC: writing—review and editing. AA and ZC: visualization. All authors contributed to the article and approved the submitted version.

## Supplementary material

### Abbreviations

AMDR, acceptable macronutrient distribution range; AMPM, automated multiple pass method; CMD, cardiometabolic disease; CVD, cardiovascular disease; HR, hazard ratio; MUFA, monounsaturated fat; NCI, national cancer institute; NCHS, national center for health statistics; NDI, national death index; NHANES, national health and nutrition examination survey; PUFA, polyunsaturated fat; SFA, saturated fat.
