# Supplementary material for: Restricted carbohydrate diets below 45% energy are not associated with risk of mortality in the National Health and Nutrition Examination Survey, 1999–2018
Source: Front Nutr. 2024 Feb 5;11:1225674. doi: 10.3389/fnut.2024.1225674 (PMC10875006; doi:10.3389/fnut.2024.1225674)
Supplement: Supplementary file 1 [file Data_Sheet_1.PDF]

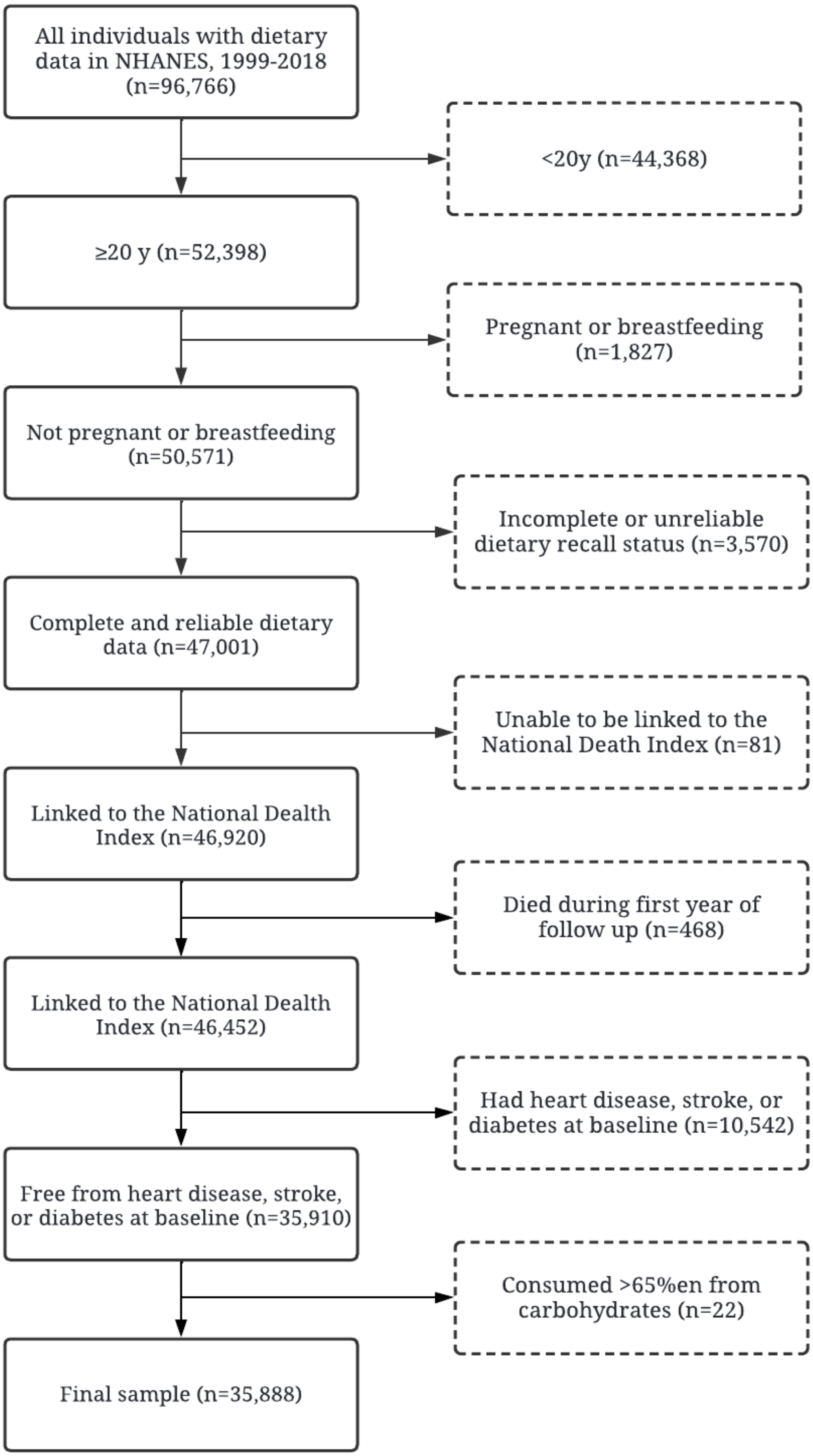

Supplemental Figure 1: Participant flowchart

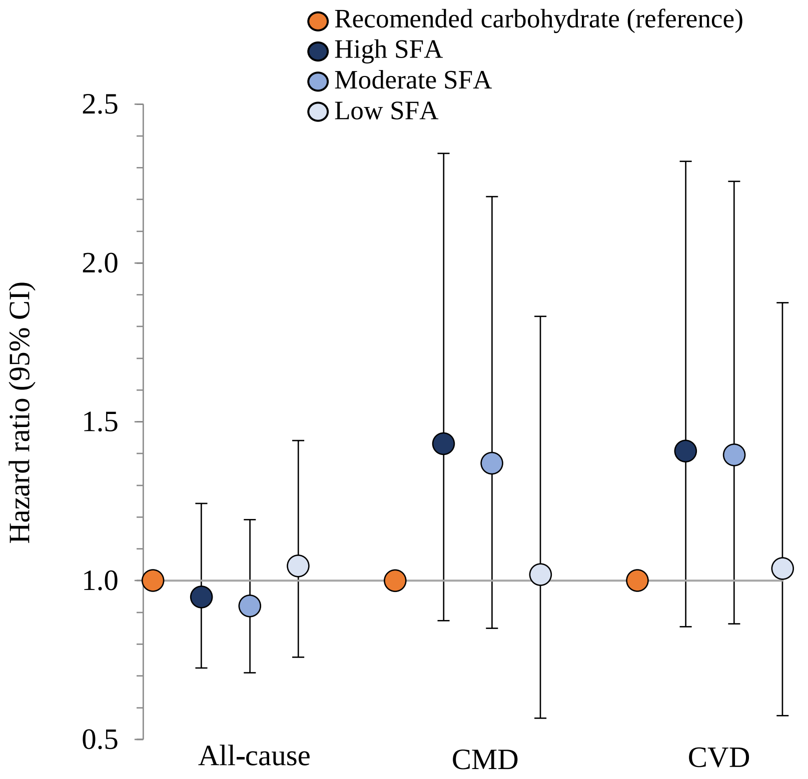

Supplemental Figure 2: Associations between carbohydrate restriction, stratified by SFA intake, and risk of mortality from all causes, cardiometabolic diseases (CMD), and cardiovascular diseases (CVD), 1999-2018 (n=35,888)

Hazard ratios (with 95% confidence intervals) comparing risk of mortality between carbohydrate restricted diet patterns (<45%en) to recommended carbohydrate diet patterns (45-65%en), calculated using Cox proportional hazards models. Models adjusted for steric acid intake (grams), age (y), sex, race/ethnicity, education, smoking status, income-to-poverty ratio, physical activity (level), baseline hypertension, baseline dyslipidemia, family history of heart disease, family history of diabetes, body mass index (kg/m2), NHANES survey wave, energy (kcal), refined grains (ounce-equivalents), added sugars (tsp equivalent), fiber (grams), protein (%en), alcohol (%en), MUFA (%en), and PUFA (%en).
CMD, cardiometabolic disease; CVD, cardiovascular disease; NHANES, National Health and Nutrition Examination Survey; SFA, saturated fat; MUFA, monounsaturated fat; PUFA, polyunsaturated fat.

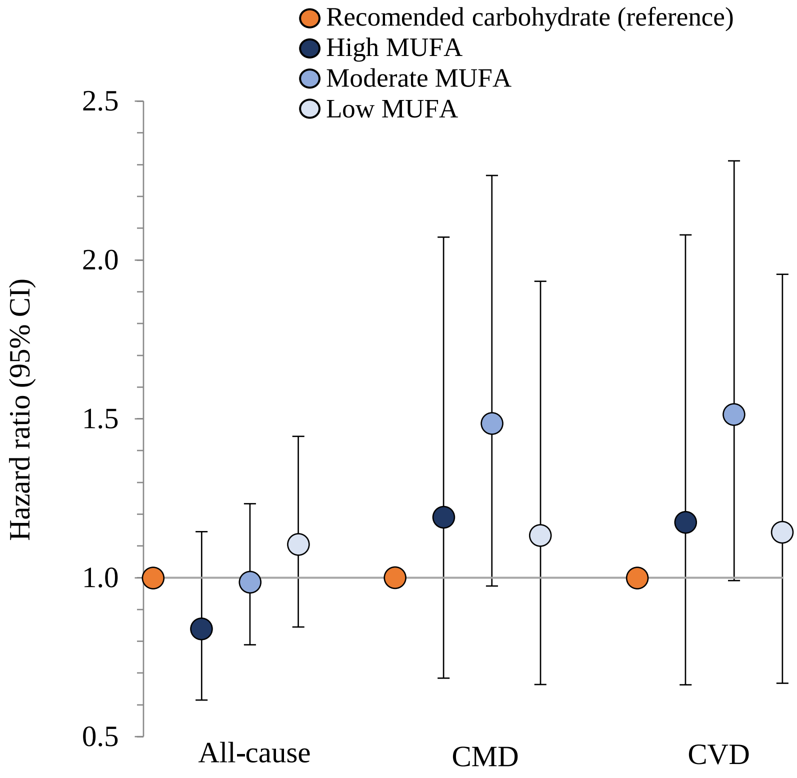

Supplemental Figure 3: Associations between carbohydrate restriction, stratified by MUFA intake, and risk of mortality from all causes, CMD, and CVD, 1999-2018, with adjustment for oleic acid (n=35,888)

Hazard ratios (with 95% confidence intervals) comparing risk of mortality between carbohydrate restricted diet patterns (<45%en) to recommended carbohydrate diet patterns (45-65%en), calculated using Cox proportional hazards models. Models adjusted for oleic acid intake (grams), age (y), sex, race/ethnicity, education, smoking status, income-to-poverty ratio, physical activity (level), baseline hypertension, baseline dyslipidemia, family history of heart disease, family history of diabetes, body mass index (kg/m2), NHANES survey wave, energy (kcal), refined grains (ounce-equivalents), added sugars (tsp equivalent), fiber (grams), protein (%en), alcohol (%en), SFA (%en), and PUFA (%en).
CMD, cardiometabolic disease; CVD, cardiovascular disease; NHANES, National Health and Nutrition Examination Survey; MUFA, monounsaturated fat; SFA, saturated fat; PUFA, polyunsaturated fat.

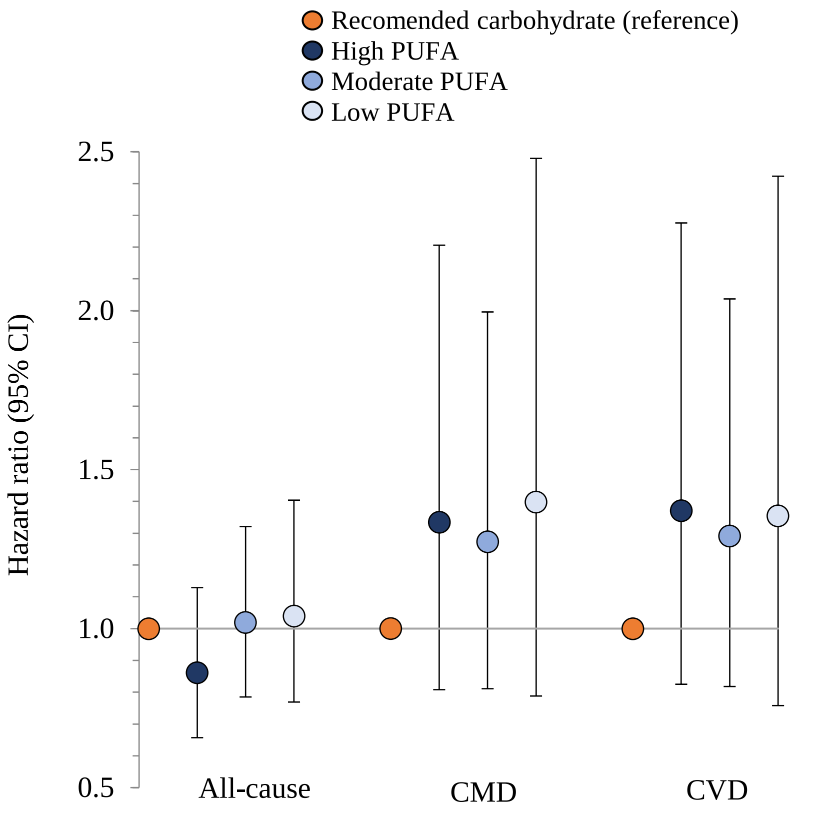

Supplemental Figure 4: Associations between carbohydrate restriction, stratified by PUFA intake, and risk of mortality from all causes, CMD, and CVD, 1999-2018, with adjustment for EPA and DHA (n=35,888)

Hazard ratios (with 95% confidence intervals) comparing risk of mortality between carbohydrate restricted diet patterns (<45%en) to recommended carbohydrate diet patterns (45-65%en), calculated using Cox proportional hazards models. Models adjusted for eicosapentaenoic acid intake (grams), docosahexaenoic acid intake (grams), age (y), sex, race/ethnicity, education, smoking status, income-to-poverty ratio, physical activity (level), baseline hypertension, baseline dyslipidemia, family history of heart disease, family history of diabetes, body mass index (kg/m2), NHANES survey wave, energy (kcal), refined grains (ounce-equivalents), added sugars (tsp equivalent), fiber (grams), protein (%en), alcohol (%en), SFA (%en), and MUFA (%en).
CMD, cardiometabolic disease; CVD, cardiovascular disease; EPA, Eicosapentaenoic acid; DHA, Docosahexaenoic acid; NHANES, National Health and Nutrition Examination Survey; PUFA, polyunsaturated fat; SFA, saturated fat; MUFA, monounsaturated fat.
